# Transplantation of mesenchymal stem cells ameliorates systemic lupus erythematosus and upregulates B10 cells through TGF-β1

**DOI:** 10.1186/s13287-021-02586-1

**Published:** 2021-09-25

**Authors:** Wang Chun, Jilai Tian, Ying Zhang

**Affiliations:** 1 Department of Rheumatology and Immunology, Nanjing Drum Tower Hospital Clinical College of Traditional Chinese and Western Medicine, Nanjing University of Chinese Medicine, Nanjing, 210008 China; 2 Department of Biochemistry and Molecular Biology, School of Medicine and Holistic Integrative Medicine, Nanjing University of Chinese Medicine, Nanjing, 210023 China

**Keywords:** Systemic lupus erythematosus (SLE), Umbilical cord mesenchymal stem cells (UC-MSCs), Regulatory B cells (Bregs), Transforming growth factor (TGF)-β1

## Abstract

**Background:**

Considerable experimental and clinical evidences have proved that human umbilical cord mesenchymal stem cells (UC-MSCs) transplantation was powerful in systemic lupus erythematosus (SLE) treatment. MSCs could upregulate regulatory B cells (Bregs) in the mice model of the other immune disease. However, the regulation of MSCs on Bregs in SLE environment remains unclear.

**Methods:**

To assess the abilities of UC-MSCs to treat SLE, MSCs were transferred intravenously to 17- to 18-week-old MRL/lpr mice. Four weeks later, mice were sacrificed. Survival rates, anti-dsDNA antibodies and renal histology were evaluated. CD4^+^ T helper (Th) cell subgroups and interleukin (IL)-10^+^ Bregs (B10) in the spleen were quantitated by flow cytometry. The changes of transforming growth factor (TGF)-β1, IL-6 and indoleamine 2,3-dioxyenase (IDO) mRNAs expressed by MSCs after co-cultured with B cells were detected using real-time polymerase chain reaction (RT-PCR). MSCs were infected by lentivirus carrying TGF-β1 shRNAs, then MSCs with low expression of TGF-β1 were conducted for co-culture in vitro and transplantation experiments in vivo.

**Results:**

UC-MSCs transplantation could efficiently downregulate 24 h proteinuria and anti-dsDNA antibodies, correct Treg/Th17/Th1 imbalances and increase the frequency of B10 cells. The expression of TGF-β1 in MSCs was significantly increased after co-culture with B cells. Downregulation of TGF-β1 in MSCs could significantly attenuate the upregulation of B10 by MSCs in vitro and in vivo. Downregulation of TGF-β1 also compromised the immunomodulation effects of MSCs on Th17 and Treg cells and the therapeutic effects of MSC transplantation.

**Conclusions:**

UC-MSCs could protect against SLE in mice and upregulate IL-10^+^ Bregs via TGF-β1.

## Introduction

Systemic lupus erythematosus (SLE), characterized by immune cell hyper-activity and accumulated autoantibodies, is a chronic autoimmune disease, which often results in multiple organ damages. Besides of the imbalance of T helper (Th)1/Th17/ regulatory T cells (Treg), the role of regulatory B cells (Bregs) is indispensable in pathogenesis of the SLE [1]. Bregs, a newly designated subset of B cells, have various phenotypes, including B1 B cells, B2 B cells and plasmocytes [2]. Bregs are characterized by producing three suppressive cytokines IL-10, TGF-β and IL-35. The IL-10-producing Bregs, named B10 cells, are currently one of the most intensively studied. Nowadays, both quantitative and qualitative deficiencies of Bregs are found in SLE patients, which are correlated with SLE disease activity index [3–6]. Low-dose CD20 mAb treatment initiated in 4-week-old mice hastened SLE disease onset, which paralleled depletion of IL-10-producing Bregs subset [7]. CD19^−/−^ NZB/W mice, which lacks CD1d^hi^CD5^+^ Bregs subset, as “B10 cells,” has significantly more severe nephritis and shorter time survive compared with the wild-type mice. Moreover, transfer of splenic CD1d^hi^CD5^+^ B cells from wild-type NZB/W mice into CD19^−/−^ NZB/W recipients is reported to prolong their survival significantly [8]. Therefore, IL-10-producing Bregs are demonstrated to play protective roles during lupus progression.

Mesenchymal stem cells (MSCs), derived from a variety of tissues such as bone marrow (BM), umbilical cords (UC), adipose tissue (AD), placenta and dental pulp, are fibroblast-like multipotent cells. They can regulate various immune cells, such as T and B lymphocytes, dendritic cells (DC) and natural killer (NK) cells [9]. During the past 10 years, considerable experimental and clinical evidences have proved that human umbilical cord mesenchymal stem cells (UC-MSCs) transplantation was potent in the treatment of SLE, especially in reducing autoantibody levels and disease activity [10, 11]. Besides, infusion of MSCs was recently reported to increase the frequency of B10 cells in experimental autoimmune encephalomyelitis (EAE) mice and trinitrobenzenesulfonic acid-induced colitis mice [12–14]. The fact that B1 cells, immature B cells and mature B cells can function as Breg cells strongly suggests that Bregs are not a definite subset and their phenotypes are altered by certain factors from the microenvironment [2]. However, the regulation of MSCs on Bregs in SLE environment remains unclear.

In this study, the changes of Bregs and CD4^+^T cells subsets were detected in MRL/lpr mice treated with UC-MSCs. Furthermore, the possible molecular mechanisms by which MSCs regulate Bregs were explored in vitro and in vivo.

## Methods

### Animals

MRL/lpr mice (female, 17- to 18-week-old) were purchased from Shanghai SLAC Laboratory Animal Co., Ltd (Shanghai, China) and kept in specific-pathogen-free (SPF) conditions in the animal center of Nanjing Drum Tower Hospital. All the animal experiments were performed under protocols approved by the Ethics Committee for Animal Research in Nanjing Drum Tower Hospital.

### Isolation and culture of UC-MSCs

UC-MSCs were obtained from Beike Biotechnology (Jiangsu, China). UC-MSCs were obtained as previously described [15] and cultured with DMEM/F12 supplemented with 10% fetal bovine serum (FBS) and 1% penicillin/streptomycin (GIBCO, Invitrogen Inc., Carlsbad CA, USA). MSCs used in this study were within passage 3–5.

### Generation of MSCs with low expression of TGF-β1

The TGF-β1 shRNA and a scrambled sequence as a control were synthesized by GenePharma (Shanghai, China) and inserted into the lentiviral vector pGIPZ (Open Biosystems, Huntsville, Alabama, USA). The sequences were as follows: TGF-β1 shRNA1 GCGGCAGCTGTACATTGACTT. TGF-β1 shRNA2 GCAACAATTCCTGGCGATACC. Control sequence TTCTCCGAACGTGTCACGT. The resulting recombinant plasmid pGIPZ-TGF-β1 shRNAs were confirmed by restriction endonuclease analysis and DNA sequencing. HEK-293 cells were co-transfected with pGIPZ-TGF-β1 shRNAs and packaging plasmids pLP1, pLP2 and pLP/VSVG (Invitrogen, Carlsbad, CA, USA). The supernatant containing lentiviral particles was harvested to determine the virus titer and used to infect MSCs. The expression of TGF-β1 was determined using real-time PCR and ELISA.

### Intravenous transplantation of UC-MSCs

UC-MSCs were collected and washed with phosphate buffered saline (PBS) three times. Cells were resuspended in PBS and intravenously infused into 17–18-week-old MRL/lpr mice at 2 × 10^5^ per 10 g body weight. Age-matched MRL/lpr mice receiving PBS were used as controls. To evaluate the effect of MSCs with TGF-β1 low expression, 17-week-old MRL/lpr mice were transplanted with MSC low expression of TGF-β, or MSC control. All mice were sacrificed at the age of 21–22 weeks to evaluate the therapeutic effects of MSC transplantation. 24-h urine was collected 1 day before sacrificed, and proteinuria was measured by Coomassie Brilliant Blue.

### Pathology assessment of kidneys

Kidneys were collected when the mice were sacrificed at 4 weeks after MSCs treatment. One half of each kidney was fixed with 4% paraformaldehyde in PBS, then embedded in paraffin and sectioned (4 μm). The sections were stained with hematoxylin and eosin (H&E) or periodic acid–Schiff (PAS) stain. The slides were examined in a blinded fashion and graded for glomerular pathology and tubulointerstitial lesion according to the grading scheme, which was reported by Chan and Kikawada [16].

### Immunologic analysis of T cell subsets in the spleen

Spleen of the mouse was grinded, isolated through a 50 μm filter (BD Biosciences, Franklin Lakes, NJ, USA), and red blood cells were lysed in a laminar flow cabinet. After washed by PBS, lymphocytes were then suspended at a density of 10^7^ cells/mL in RPMI 1640 complete medium, which was supplemented with 100 U/mL penicillin, 100 μg/mL streptomycin, 2 mmol/L glutamine and 10% heat-inactivated fetal calf serum (GIBCO). To identify Tregs, 5 × 10^5^ lymphocytes were labeled with FITC-labeled anti-CD4 and allophycocyanin (APC)-cyanine (Cy)7-labeled Foxp3. For Th1/Th2/Th17 cell subgroup analyses, 2 × 10^6^ cells were stimulated with 50 ng/mL phorbol 12-myristate 13-acetate (PMA) plus 1 μg/mL ionomycin and 5 μg/mL brefeldin A at 37 °C for 4 h. Then, the staining was performed with FITC-anti-CD4, APC-anti-IFN-γ (Th1), PE-anti-IL4 and (Th2) and APC-anti-IL17A (Th17). All antibodies were purchased from eBioscience (San Diego, CA, USA). Data were acquired by flow cytometer (Calibur, BD Biosciences, CA, USA) and analyzed with FlowJo software (Tree Star, USA).

### Immunologic analysis of B10 cells in the spleen

Lymphocytes of the spleen were isolated as described above. The B cell was isolated according to the protocol of B Cell Isolation Kit (Miltenyi Biotec, Bergisch Gladbach, Germany) by depletion of non-B cells. 5 × 10^6^ B cells/mL was resuspended in RPMI 1640 and was stimulated for 48 h with combinations of recombinant murine sCD40 Ligand (PeproTech, at 1 μg/ml) and CpG (InvivoGen ODN 1826, at 4 μg/ml). 50 ng/mL phorbol 12-myristate 13-acetate (PMA) plus 1 μg/mL ionomycin and 5 μg/mL brefeldin A were added for the last 4 h. Then, cell staining was performed with FITC-anti-CD19 and PE-anti-IL10 (eBioscience). Data were acquired by flow cytometer and analyzed with FlowJo software.

### Co-culture of UC-MSCs and B cells

UC-MSCs were cultured overnight to allow adherence at concentration of 1.25 × 10^4^ cells/cm^2^ in complete DMEM/F12 medium. B cells were co-cultured with or without MSCs at a ratio of 10:1 in RPMI 1640 and stimulated for 48 h with combinations of recombinant murine sCD40 Ligand (PeproTech, at 1 μg/ml) and CpG (InvivoGen ODN 1826, at 4 μg/ml). 50 ng/mL phorbol 12-myristate 13-acetate (PMA) plus 1 μg/mL ionomycin and 5 μg/mL brefeldin A were added at the last 4 h. Then MSCs and B cells were collected for RNA isolation and flow cytometry analysis, respectively. To evaluate the effect of MSCs with TGF-β1 low expression, B cells were co-cultured with MSCs low expression of TGF-β1, or MSCs control. Then both MSCs with low expression of TGF-β1 and control were collected for RNA isolation. The flow cytometry analysis to detect Breg was described above.

### Quantitative real-time polymerase chain reaction (PCR) and ELISA

RNA was isolated from UC-MSCs using a total RNA isolation reagent (Vazyme Biotech), and cDNA was transcribed from RNA with HiScript® II Q RT SuperMix (Vazyme Biotech). The transcript levels of transforming growth factor (TGF)-β1, interleukin (IL)-6 and indoleamine 2,3-dioxyenase (IDO) levels were determined by StepOne Plus Real-Time PCR System (Applied Biosystems) with AceQ® qPCR SYBR® Green Master Mix (High ROX Premixed, Vazyme Biotech). Data were analyzed using 2^−ΔΔCT^ method, normalizing to the expression of glyceraldehyde 3-phosphate dehydrogenase (GAPDH). The primer sequences used were as follows: GAPDH, forward, 5′-GCACCGTCAAGGCTGAGAAC-3′, reverse, 3′-TGGTGAAGACGCCAGTGGA-5′. TGF-β1, forward, 5′-AGCGACTCGCCAGAGTGGTTA-3′, reverse 5′-GCAGTGTGTTATCCCTGCTGTCA-3′. IL-6, forward, 5′-AAGCCAGAGCTGTGCAGATGAGTA-3′, reverse, 5′-TGTCCTGCAGCCACTGGTTC-3′. IDO, forward, 5′-GAATGGCACACGCTATGGAA-3′, reverse, 5′-CAGACTCTATGAGATCAGGCAGATG-3′. IL-10, forward, 5′-GAGATGCCTTCAGCAGTGAGTGAAGA-3′, reverse, 5′-AGTTCACATGCGCCTTGATGTC-3′. We detected the amounts of TGF-β1 in the conditioned media with ELISA Kits (eBioscience) according to the manufacturer’s instructions.

### Statistical analysis

All data were expressed as the mean ± SD. *t* test, Kruskal–Wallis and Mann–Whitney *U* test were used to assess the significance between group comparisons using SPSS 22.0 software. Kaplan–Meier analysis was conducted to compare the survival status between the two groups. *P* values less than 0.05 were considered statistically significant.

## Results

### UC-MSC transplantation could alleviate disorders and upregulate B10 cells in SLE mice

**Fig. 1:**
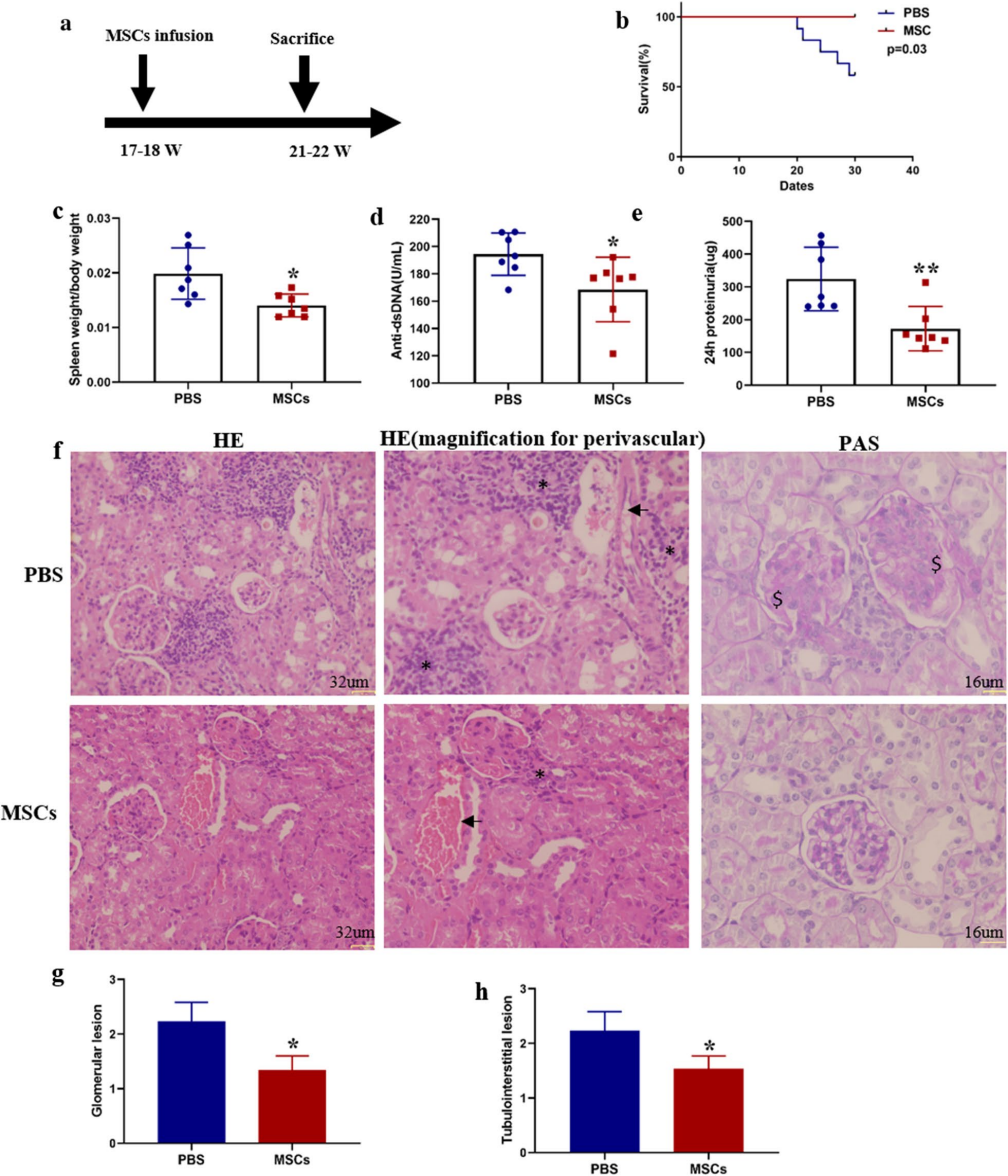
Transplantation of UC-MSCs relieves SLE phenotypes in MRL/lpr mice. **a** A scheme of UC-MSC transplantation procedures; **b** the survival status of MRL/lpr mice; **c** Spleen index of MRL/lpr mice after MSCs therapy; **d** Serum anti-dsDNA antibodies of MRL/lpr mice; **e** 24-h urinary protein (μg) post-MSCs transplantation. **f** Representative images of renal interstitial inflammatory cell infiltration (HE original magnification ×200) and aberrant mesangial, cell proliferation (PAS original magnification ×400); “*” represents interstitial inflammatory cell infiltration, the arrow “ → ” indicates vessels, and “$” means mesangial and cell proliferation. **g** The scores of glomerular pathologies; **h** Interstitial inflammation scores. Data are shown in “mean ± SD.” For **b**–**e** PBS (*n* = 12, death *n* = 5), UC-MSCs (*n* = 7, death *n* = 0). For **f**–**h** PBS (*n* = 7), UC-MSCs (*n* = 7). *p < 0.05, **p < 0.01. MSCs, umbilical cord mesenchymal stem cells; PBS, phosphate buffered saline

**Fig. 2:**
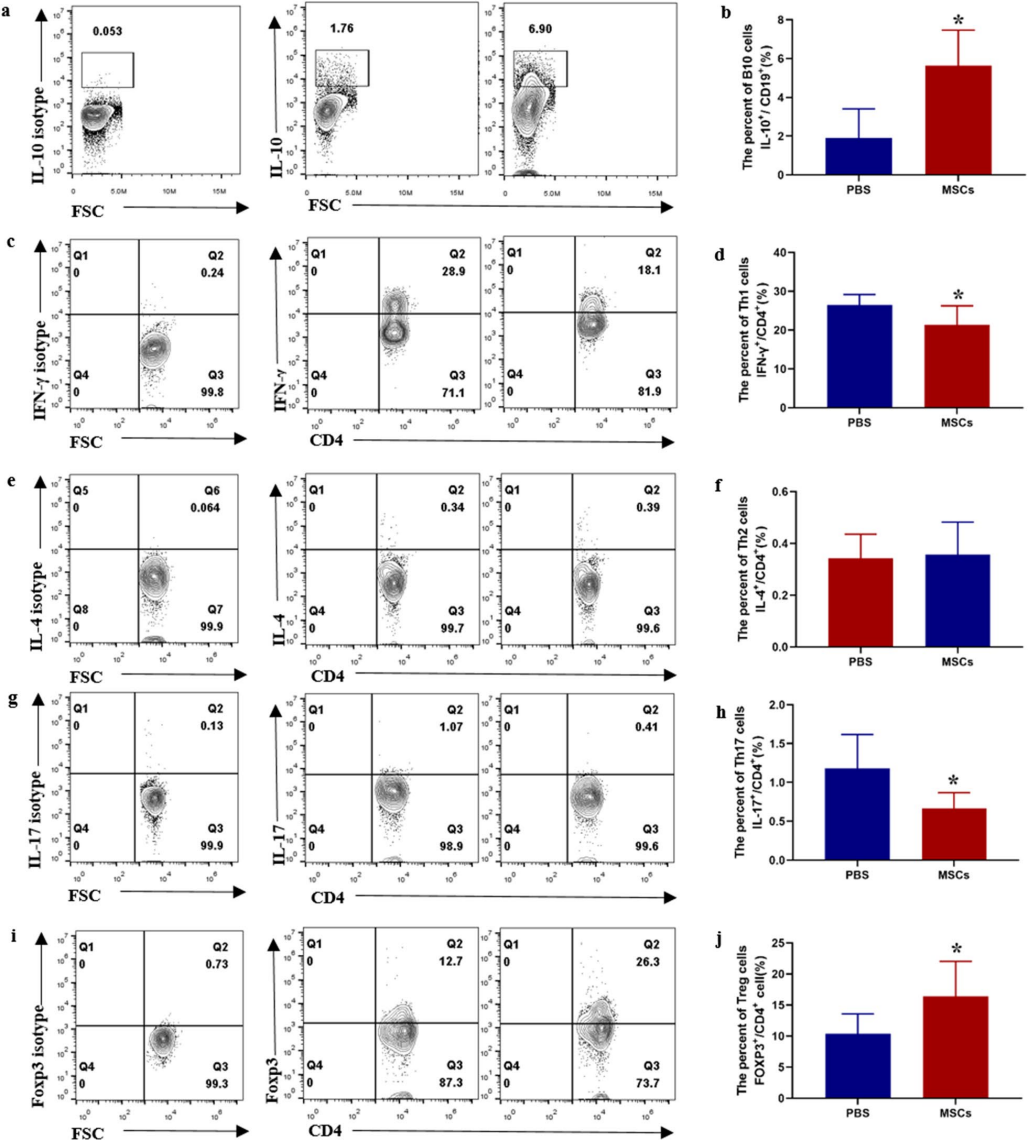
Flow cytometric analysis of B10, Th1, Th2, Th17 and Treg cells in the spleens of MRL/lpr mice after MSCs transplantation. **a**, **c**, **e**, **g**, **i** Representative FACS images of CD19^+^IL-10^+^ (B10 cells), CD4^+^IFN-γ^+^ (Th1 cells), CD4^+^IL4^+^ (Th2 cells), CD4^+^IL17^+^ (Th17 cells) and CD4^+^Foxp3^+^ (Treg cells). For B10 cells detection, B cells were isolated from the spleen of mice and were stimulated for 48 h with sCD40 Ligand and CpG. **b**, **d**, **f**, **h**, **j** Percentages of B10, Th1, Th2, Th17 and Treg cells in the spleens of MRL/lpr mice with or without MSCs transplantation. Data are mean ± SD. PBS (*n* = 7), MSCs (*n* = 7). *p < 0.05. PBS, phosphate buffered saline; MSCs, umbilical cord mesenchymal stem cells

Eighteen-week MRL/lpr mice were employed to evaluate the therapeutic effects of UC-MSCs in SLE. 2 × 10^5^ MSCs per 10 g were injected through tail vein, and the mice were sacrificed 4 weeks later (Fig. 1a). MRL/lpr mice with UC-MSCs transplantation displayed a significant improvement in the survival status (Fig. 1b) and a dramatically decrease in spleen index (spleen weight/body weight g/g) (Fig. 1c). As known, autoantibodies play a crucial role in the pathogenesis of SLE. UC-MSC transplantation also resulted in a significant reduction in serum anti-dsDNA antibody (Fig. 1d). Next, whether treatment with MSCs can reduce the renal injury in SLE mice was investigated. Less proteinuria at 22 weeks was found in the group treated with UC-MSCs (Fig. 1e). We then assessed the histopathological changes in kidneys of MRL/lpr mice received UC-MSCs. Results showed that, MSC transplantation significantly improved glomerular capillary cell proliferation and reduced renal interstitial inflammatory cell infiltration (Fig. 1f–h). Finally, we examined the changes of Th1, Th2, Th17, Treg and B10 in the spleen of MRL/lpr mice 4 weeks after MSC transplantation. As shown in Fig. 2, MSCs infusion significantly increased the frequency of B10 cell in B cells (Fig. 2a, b). Meanwhile, CD4^+^ T cells producing IFN-γ (Th1) and IL17 (Th17) significantly decreased, and Foxp3^+^ Treg cells increased in MRL/lpr mice treated with UC-MSCs (Fig. 2c, d, g–j). UC-MSCs transplantation did not affect the frequency of Th2 cells (Fig. 2e, f).

### TGF-β1 produced by UC-MSCs mediates the upregulation of B10 cells

**Fig. 3:**
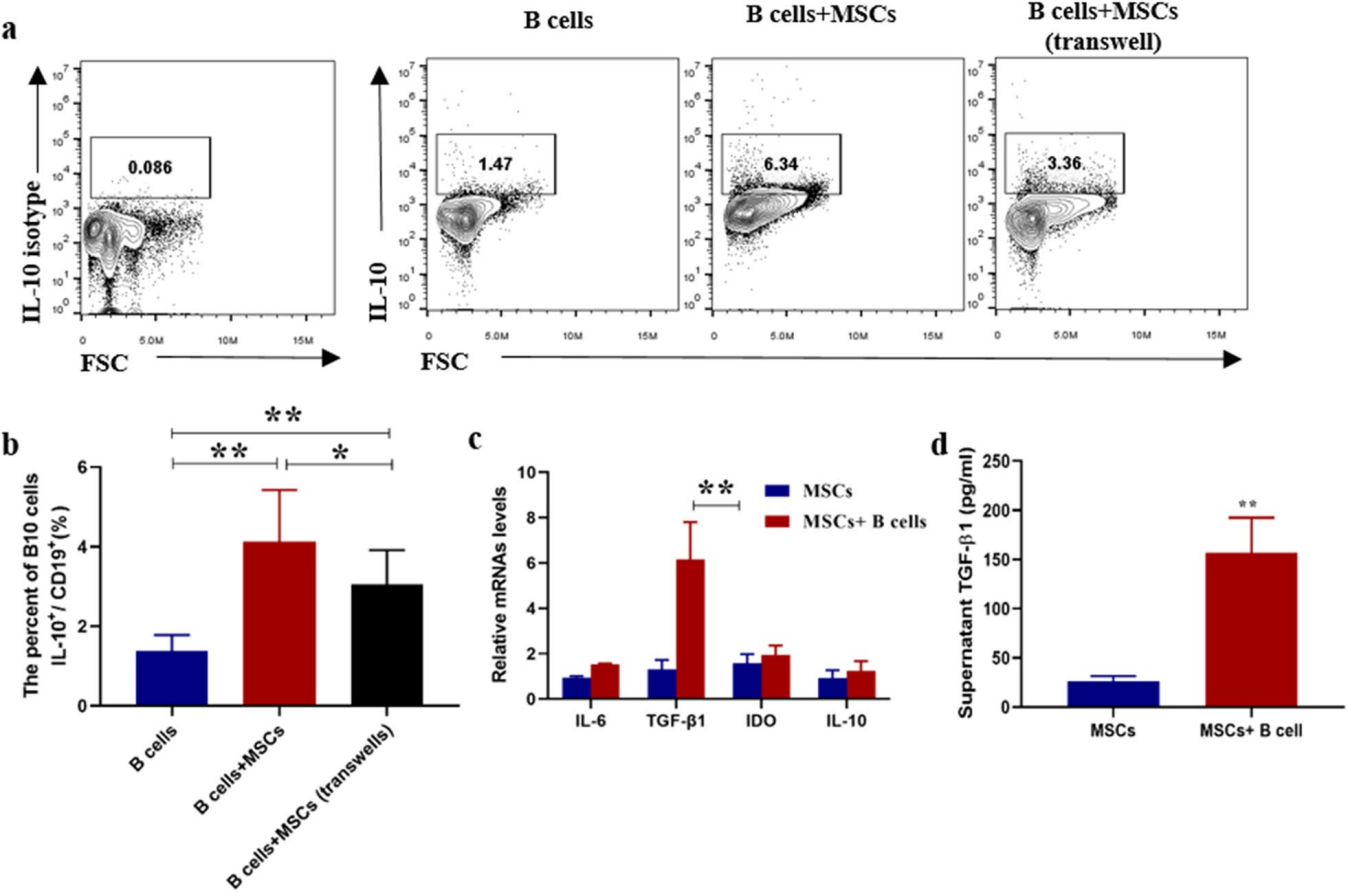
UC-MSCs up-regulated B10 and produced functional factors after co-cultured with B cells in vitro. **a** Representative FACS images of CD19^+^IL-10^+^ (B10 cells), **b** percentages of B10 in B cells after co-cultured with MSCs directly or indirectly. **c** The mRNA of IL-6, IL-10, TGF-β1 and IDO expressed by MSCs co-cultured with or without B cells was analyzed by real-time PCR. **d** TGF-β1 in supernatant were significantly increased after co-culture. Data are mean ± SD. B cells (*n* = 6), B cells + MSCs, B cells and MSCs were co-cultured directly (*n* = 6), B cells + MSCs (transwell), B cells and MSCs were co-cultured indirectly (*n* = 6). Data are mean ± SD. *p < 0.05, **p < 0.01

**Fig. 4:**
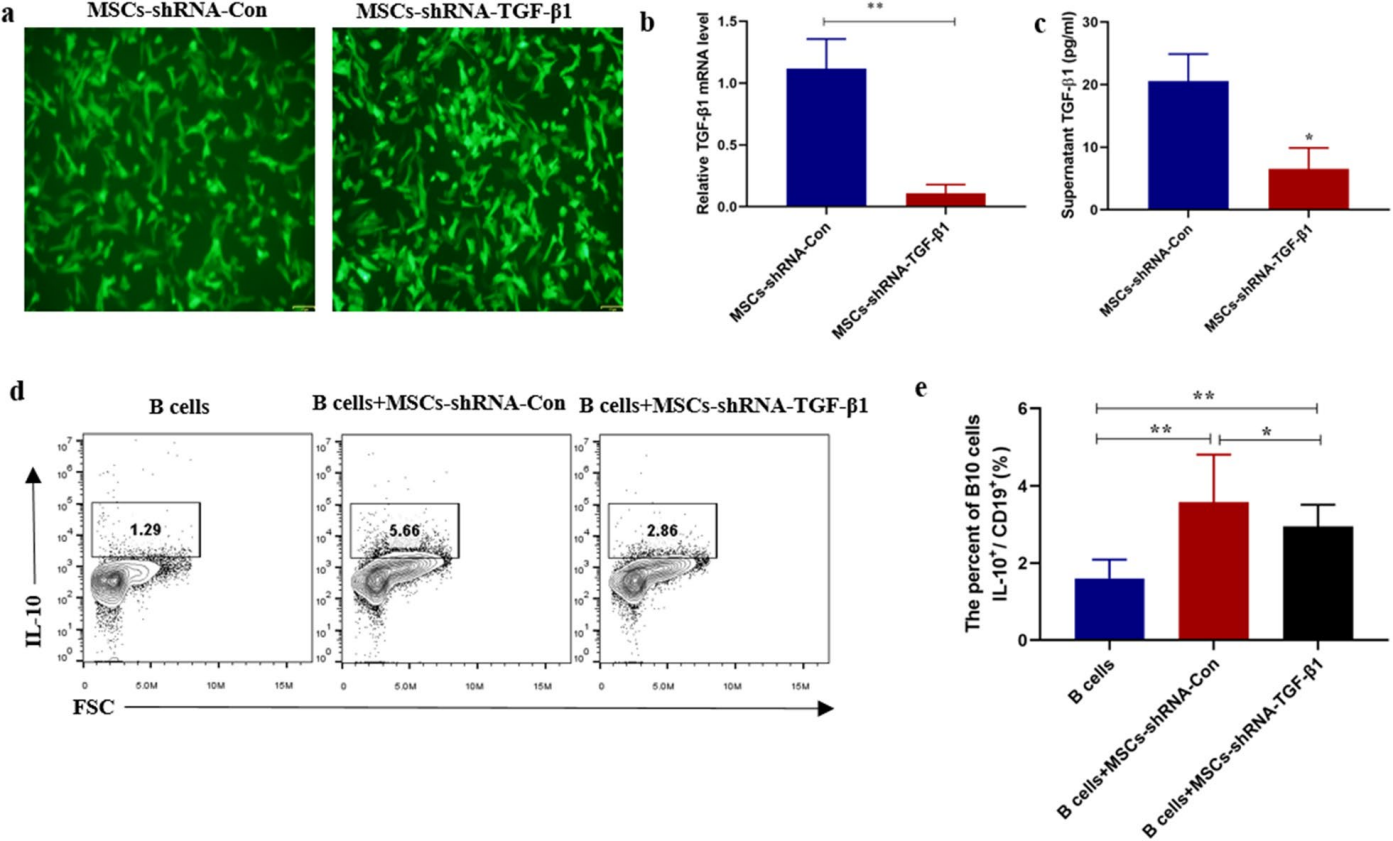
Knockdown of TGF-β1 by shRNA abolished the upregulation of B10 cell by MSCs. **a** Fluorescence microscope showed MSCs infected by lentivirus carrying TGF-β1 shRNA and Control shRNA for 48 h. TGF-β1 interference efficiency was determined by real-time PCR (**b**) and ELISA (**c**). **d** Representative FACS images of B10 cells and **e** percentages of B10 in B cells after co-cultured with MSCs low expression of TGF-β1. Data are mean ± SD. B cells (*n* = 6), B cells + MSCs-shRNA-Con, B cells co-cultured with MSCs infected by control lentivirus (*n* = 6), B cells + MSCs-shRNA-TGF-β1, B cells co-cultured with MSCs infected by lentivirus with TGF-β1 shRNA (*n* = 6). *p < 0.05, **p < 0.01

We next investigated which factor mediated the regulation of B10 by MSCs in SLE. First, UC-MSCs and B cells were co-cultured either directly or indirectly (using transwell). After 48 h, MSCs significantly increased the percentage of B10 in the direct co-culture system, while the indirect groups significantly attenuated the upregulation of B10 by MSCs (Fig. 3a, b). Because the current reported factors mostly include TGF-β, IL-6 and IDO [17], we next detected the changes of these mRNAs in MSCs after co-cultured with B cells. As shown in Fig. 3c, d, TGF-β1 mRNA and supernatant TGF-β1 in the condition were significantly increased after co-culture. To confirm whether TGF-β1 contributed to the upregulation of B10 cells, MSCs with low expression of TGF-β1 were constructed by infected with lentivirus carrying TGF-β1 shRNAs, and the low expression of TGF-β1 was confirmed by real-time PCR (Fig. 4a, b) and ELISA (Fig. 4c). We found that TGF-β1 could significantly abrogate the upregulation of B10 cells by MSCs (Fig. 4c, d).

### TGF-β1 downregulation attenuates the therapeutic effects of MSC transplantation in MRL/lpr mice

**Fig. 5:**
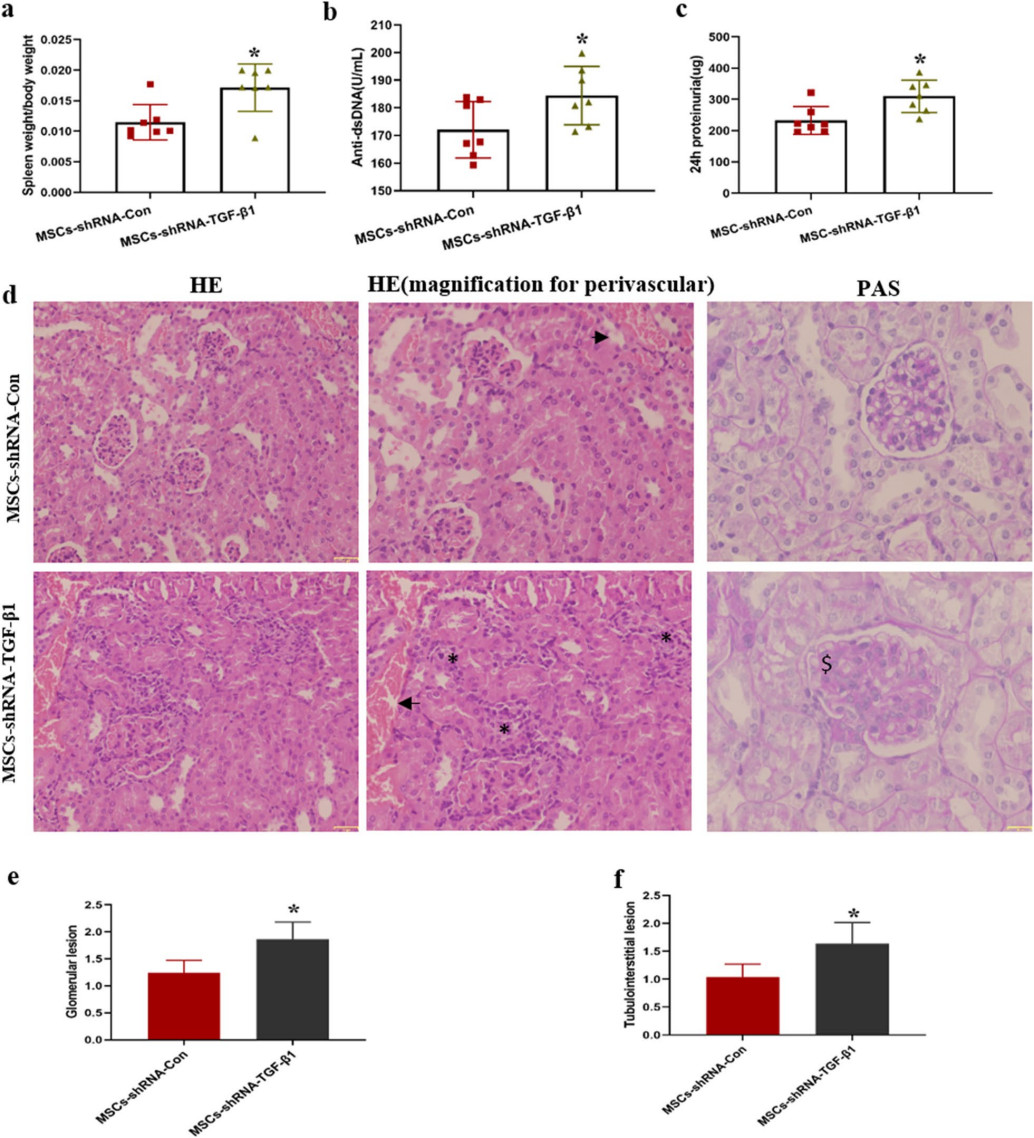
TGF-β1 downregulation weakened the therapeutic effect of MSC transplantation in MRL/lpr mice. Spleen index (**a**), serum anti-dsDNA antibodies (**b**), 24 h urinary protein (**c**) of MRL/lpr mice treated with MSCs-shRNA-TGF-β1 transplant. **d** Representative images of glomerular capillary cell proliferation and renal interstitial inflammatory cell infiltration of MRL/lpr mice treated with MSCs-shRNA-TGF-β1 transplant; “*” represents interstitial inflammatory cell infiltration, the arrow “ → ” indicates vessels, and “$” means mesangial and cell proliferation. The scores of glomerular pathologies (**e**) and interstitial inflammation scores (**f**) of MRL/lpr mice treated with MSCs-shRNA-TGF-β1 transplant. Data are mean ± SD. MSCs-shRNA-Con (*n* = 7), MSCs-shRNA-TGF-β1 (*n* = 7). *p < 0.05, **p < 0.01. MSCs-shRNA-Con, MSCs infected by control lentivirus; MSCs-shRNA-TGF-β1, MSCs infected by lentivirus with TGF-β1 shRNA

To confirm whether the therapeutic effects of MSCs were dependent on TGF-β1, 17-week MRL/lpr mice were intravenously injected MSCs control, or MSCs with TGF-β1 low expression. Four weeks later, the mice were sacrificed to evaluate the therapeutic effects of MSCs with TGF-β1 low expression. As shown in Fig. 5, MRL/lpr mice, which were transplanted UC-MSCs with TGF-β1 low expression, displayed a significant increase in spleen index (Fig. 5a) and a dramatically increase in serum anti-dsDNA antibody (Fig. 5b). Much proteinuria and heavier glomerular lesion at 21 weeks were found in the group treated with UC-MSCs expressing low TGF-β1 (Fig. 5c–e). Renal interstitial inflammatory cell infiltration was increased in the mice received UC-MSCs with low expression of TGF-β1 (Fig. 5d, f).

### TGF-β1 downregulation weakens modulation of B10 cells by MSCs

**Fig. 6:**
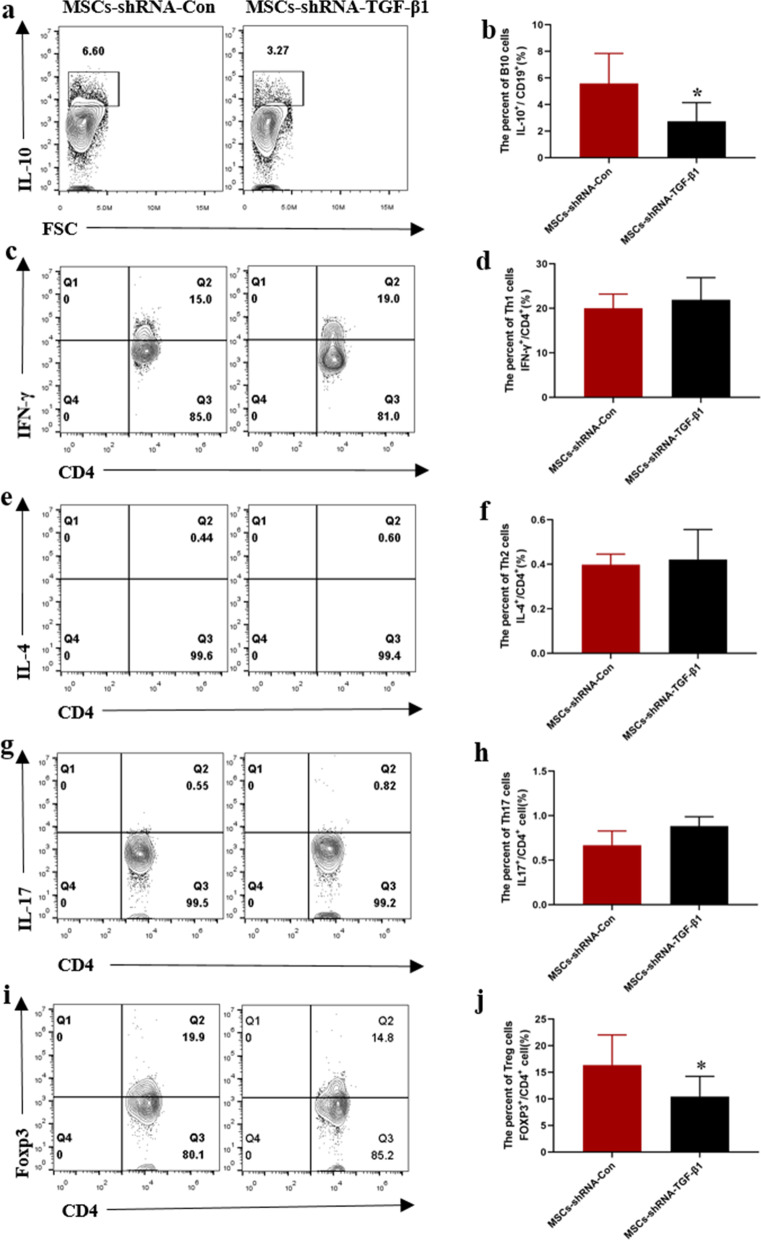
Flow cytometric analysis of B10, Th1, Th2, Th17 and Treg cells in the spleens of MRL/lpr mice treated by MSCs with TGF-β1 low expression. **a**, **c**, **e**, **g**, **i** Representative FACS images of CD19^+^IL-10^+^ (B10 cells), CD4^+^IFN-γ^+^ (Th1 cells), CD4^+^IL4^+^ (Th2 cells), CD4^+^IL17^+^ (Th17 cells) and CD4^+^Foxp3^+^ (Treg cells). For B10 cells detection, B cells were isolated from the spleens of mice and were stimulated for 48 h with sCD40 Ligand and CpG. **b**, **d**, **f**, **h**, **j** Percentages of B10, Th1, Th2, Th17 and Treg cells in the spleen of MRL/lpr mice treated by MSCs with TGF-β1 low expression. Data are mean ± SD. MSCs-shRNA-Con (*n* = 7), MSCs-shRNA-TGF-β1 (*n* = 7). *p < 0.05, **p < 0.01. MSCs-shRNA-Con, MSCs infected by control lentivirus; MSCs-shRNA-TGF-β1, MSCs infected by lentivirus with TGF-β1 shRNA

Finally, we examined whether the modulatory effects of MSCs on B10 and T cells subsets were dependent on TGF-β1 in MRL/lpr mice. As shown in Fig. 6, compared with MSC control group, knockdown of TGF-β1 in MSCs attenuated the upregulation of B10 cells in MRL/lpr mice (Fig. 6a, b). Knockdown of TGF-β1 in MSCs significantly attenuated the downregulation of Th17 cells (Fig. 6g, h), and upregulation of Foxp3^+^ Treg cells (Fig. 6i, j). TGF-β1 downregulation had no evident effects on Th1, Th2 cells compared with MSCs control group (Fig. 6c–f).

## Discussion

In the past decades, new drugs for SLE have greatly improved the efficacy and quality of life in SLE patients. However, the fight against SLE is far from over, as there is still no ideal treatment or cure. Findings from recent studies have confirmed that immunologic factors play a central role in the pathogenesis of SLE. Imbalances among CD4^+^ T cells subgroups and abnormal of Bregs were found in SLE patients. Treg/Th1, Treg/Th17 ratios and Breg are associated with disease activity and are known as potential prognostic indicators for predicting treatment efficacy.

MSCs therapy is a promising approach in several immune diseases treatment, including SLE. Our previous studies have found that UC-MSCs transplantation could alleviate experimental SLE and SLE patients and could modulate Treg/Th1/Th17 imbalances [11, 17, 18]. However, up to now, there are few reports concerning the effects of UC-MSCs on B10 cells in SLE. In this study, UC-MSCs treatment conclusively alleviated experimental SLE and boosted the proportion of B10 cells. B10, as one subset of regulatory B cells (Bregs), showed immunosuppressive function via secretion of IL-10. Although there are controversies concerning the definition and surface markers of Bregs, this subset is known to exert immunosuppressive functions by regulating Th cells and Tregs, such as inducing T cell and B cell apoptosis, and inhibiting other immune-related cells, including CD8^+^ T cells and natural killer T cells [2]. Furthermore, Bregs are associated with autoimmune diseases, such as type I diabetes, graft versus host disease (GVHD), arthritis and lupus [9]. Two studies have reported increases in the numbers of CD5^+^ B cells in EAE model and GVHD after BM-MSCs transplantation [19, 20]. However, these studies did not analyze the potential mechanisms and function of CD5^+^ B cells. In another study, the number of B10 cells was significantly elevated after UC-MSC transfer in colitis model and exerted their function specifically in the inflamed areas [14]. They conducted further in vivo and in vitro study to confirm that MSCs can regulate B10 cells to modulate the immune status of T cells, but they did not explain the mechanisms how UC-MSCs induce B10 cell differentiation [14].

Several key mechanisms of MSCs regulation B10 cells have been described. Human adipose tissue-derived MSCs induced Bregs independently of T cells [21]. MSCs enhanced the frequency and immunosuppressive capacity of B10 cells from multiple sclerosis patients via IDO in vitro [12]. In our previous study, MSCs transplantation significantly increased serum TGF-β1 levels in SLE patients [18], and the expression of TGF-β1 in MSCs was up-regulated after co-cultured with peripheral blood mononuclear cell (PBMCs) [17]. In this study, co-culture with B cells significantly increased TGF-β1 expression in MSCs, and knockdown of TGF-β1 in MSCs significantly weakened the upregulation of B10 cells in vitro and in vivo. Consistently, sirolimus could significantly amplify Bregs, which was also dependent on TGF-β1 [22].

Furthermore, our study demonstrated that knockdown of TGF-β1 in MSCs also attenuated the therapeutic effects, and the weakened effects of MSCs on amplifying Bregs might play an important role in this process. On the other hand, TGF-β contributed to the modulation of Tregs by MSCs in our previous study [17], so knockdown of TGF-β directly weakened Treg upregulation after MSC transplantation. Because Bregs could also regulate Tregs [2], knockdown of TGF-β in MSCs could weaken Treg upregulation indirectly. The impaired amplifying of Bregs and Tregs by MSCs with low expression of TGF-β might contribute to MSCs inefficient regulation on Th17 cells.

## Conclusion

Our study indicated that UC-MSCs could protect against SLE by correcting Treg/Th17/Th1 imbalances in mice. The underlying mechanism possibly acts via boosting the frequency of IL-10^+^ Bregs through producing TGF-β1.

## Data Availability

All data generated or analyzed during this study are included in this article.

## Abbreviations

SLE: Systemic lupus erythematosus
UC-MSCs: Umbilical cord mesenchymal stem cells
Bregs: Regulatory B cells
Tregs: Regulatory T cells
TGF-β1: Transforming growth factor-β1
IDO: Indoleamine 2,3-dioxyenase
IL: Interleukin
RT-PCR: Real-time polymerase chain reaction
GVHD: Graft versus host disease
PBMC: Peripheral blood mononuclear cell
